# Identification of calgranulin B interacting proteins and network analysis in gastrointestinal cancer cells

**DOI:** 10.1371/journal.pone.0171232

**Published:** 2017-02-02

**Authors:** Kyung-Hee Kim, Seung-Gu Yeo, Byong Chul Yoo, Jae Kyung Myung

**Affiliations:** 1 Omics Core Laboratory, Research Institute, National Cancer Center, Goyang-si, Gyeonggi-do, Republic of Korea; 2 Colorectal Cancer Branch, Research Institute, National Cancer Center, Goyang-si, Gyeonggi-do, Republic of Korea; 3 Department of Radiation Oncology, Soonchunhyang University College of Medicine, Cheonan, Chungnam, Republic of Korea; 4 Department of System Cancer Science, Graduate School of Cancer Science and Policy, National Cancer Center, Goyang-si, Gyeonggi-do, Republic of Korea; University of South Florida, UNITED STATES

## Abstract

Calgranulin B is known to be involved in tumor development, but the underlying molecular mechanism is not clear. To gain insight into possible roles of calgranulin B, we screened for calgranulin B-interacting molecules in the SNU-484 gastric cancer and the SNU-81 colon cancer cells. Calgranulin B-interacting partners were identified by yeast two-hybrid and functional information was obtained by computational analysis. Most of the calgranulin B-interacting partners were involved in metabolic and cellular processes, and found to have molecular function of binding and catalytic activities. Interestingly, 46 molecules in the network of the calgranulin B-interacting proteins are known to be associated with cancer and FKBP2 was found to interact with calgranulin B in both SNU-484 and SNU-81 cells. Polyubiquitin-C encoded by *UBC*, which exhibited an interaction with calgranulin B, has been associated with various molecules of the extracellular space and plasma membrane identified in our screening, including Na-K-Cl cotransporter 1 and dystonin in SNU-484 cells, and ATPase subunit beta-1 in SNU-81 cells. Our data provide novel insight into the roles of calgranulin B of gastrointestinal cancer cells, and offer new clues suggesting calgranulin B acts as an effector molecule through which the cell can communicate with the tumor microenvironment via polyubiquitin-C.

## Introduction

Calprotectin is heterotetrameric calgranulin A and B complex that were noncovalently bonded without a peptide bridge between two subunits. The amounts of calprotectin in blood or extracellular body fluids are reportedly increased under many pathological conditions, such as rheumatoid arthritis, inflammatory bowel diseases, viral infection, microbial infection, tumors, and many inflammatory conditions [1].

Various functions of calprotectin have been reported, such as stimulation of fibroblast growth and beta 2-integrin-mediated neutrophil adhesion, neutrophil chemoattraction, and macrophage deactivation [2–5]. Calprotectin is also believed to function in altering the cytoskeleton and cell shape, transducing signals, and modulating intracellular calcium.

One of the two units of calprotectin, calgranulin B, is a small calcium-binding protein that is mainly found in granulocytes, monocytes, and activated keratinocytes [6–10]. It has also emerged as a marker for non-inflammatory pathological conditions, such as tumor development. Calgranulin B is reportedly overexpressed in various tumor types, including ovarian cancer, head and neck tumors, pulmonary carcinoma, and prostate cancer [11]. In addition, it is secreted by intestinal monocytes and epithelial cells, and elevated levels of calgranulin B have been detected in stool samples from colorectal cancer patients. We previously reported that calgranulin B is a candidate fecal marker for the diagnosis of colorectal cancer [12], and more recently showed that combining the fecal occult blood test (the established means of colorectal cancer screening) with calgranulin B screening can increase the sensitivity of colorectal cancer detection [13]. However, the intracellular molecular mechanism underlying the involvement of calgranulin B in tumor development is unknown.

Here, we set out to investigate the role of calgranulin B in gastrointestinal cancer by identifying calgranulin B-interacting partners in cancer cell lines.

## Materials and methods

### Human cell lines

SNU-81 colorectal carcinoma cells, SNU-484 gastric carcinoma cells, and HEK293 human embryonic kidney cells were obtained from the Korean Cell Line Bank (KCLB, Seoul, Korea).

### Yeast two hybrid (Y2H)

The full-length cDNA of human calgranulin B was PCR amplified and cloned into the pGBKT7 vector (containing the GAL4 DNA-binding domain). The pGBKT7-calgranulin B construct did not show any autonomous transcriptional activation or cytotoxicity following transformation into the yeast strain, Y2H Gold.

SNU-484, SNU-81, and HEK293 cells were used to construct cDNA libraries in the pGADT7-Rec vector (containing a GAL4 activation domain) using Matchmaker Library Construction and Screening kits (Clontech, Santa Clara, CA, USA). Each library was then transformed into the Y187 yeast strain, and Y2H screening was performed using the Matchmaker Two-Hybrid system (Clontech). Positive clones were selected based on their ability to grow on synthetic dropout (SD) medium/-LTH/X-α-Gal (TDO). Their cDNA inserts were PCR amplified, sequenced, and subjected to BLAST alignment. Interaction between the bait and identified prey clones was verified by co-transforming the purified prey plasmid plus the bait pGBKT7-calgranulin B construct into Y2H Gold cells, and then selecting clones on SD/-LTHA/ X-α-Gal medium. Co-transformation of pGBKT7-p53 plus pGADT7-SV40 was used as a positive control, while co-transformation of pGBKT7-p53 with empty pGADT7 vector was used as a negative control.

### Gene ontology and top disease information analysis

Computational analysis was applied to all of the molecules identified as interacting with calgranulin B in the three cell lines. Gene ontology (GO) analysis of the relevant biological processes, cellular components, and molecular functions was performed using the Protein Analysis Through Evolutionary Relationships program (PANTHER, www.pantherdb.org), which refers to a curated database of protein families, functions and pathways [14,15]. GO terms assigned into identified molecules were classified according to their function.

The most highly represented diseases and disorders information was obtained from Ingenuity Pathway Analysis (IPA, www.quiagen.com/ingenuity), which determines functions of identified molecules from literature-based information.

### Protein class and pathway analysis

Furthermore, the protein class levels and pathways of all identified molecules were assessed using the PANTHER pathway program, which refers to a database containing: 165 expertly curated metabolic and signaling pathways; 20,851 proteins directly associated with these pathways, along with an evidence code; and 3569 distinct literature references (http://pid.nci.nih.gov/2009/090414/full/pid.2009.1.shtml).

### Molecular interaction and network analysis

IPA, which determines the interactions and functions of identified gene products based on the information available in the literature, was used to map the identified molecules with respect to their interconnections. IPA scans input molecules to identify networks using the Ingenuity Pathways Knowledge Base (IPKB) for interaction between identified molecules. Molecular interactions are shown by grey lines, the identified calgranulin B-interacting molecules are highlighted in purple, and molecules that have been associated with cancer are indicated by dotted blue lines.

STRING analysis (https://www.string-db.org) was used to find direct and indirect interactions between all identified molecules interacting with calgranulin B in the extracellular space and plasma membrane. STRING is a database which provides direct and indirect associations from computational prediction, and interactions from other databases for interaction analysis [16,17]. STRING score is calculated from the combination of all predictions and network analysis was set at a medium confidence. Eight different colored lines were used to represent the types of evidence for associations, as follows: green, neighborhood evidence; red, gene fusion; blue, co-occurrence; black, co-expression; purple, experimental; light blue, database; yellow, text mining; sky blue, protein homology.

## Results

**Fig 1:**
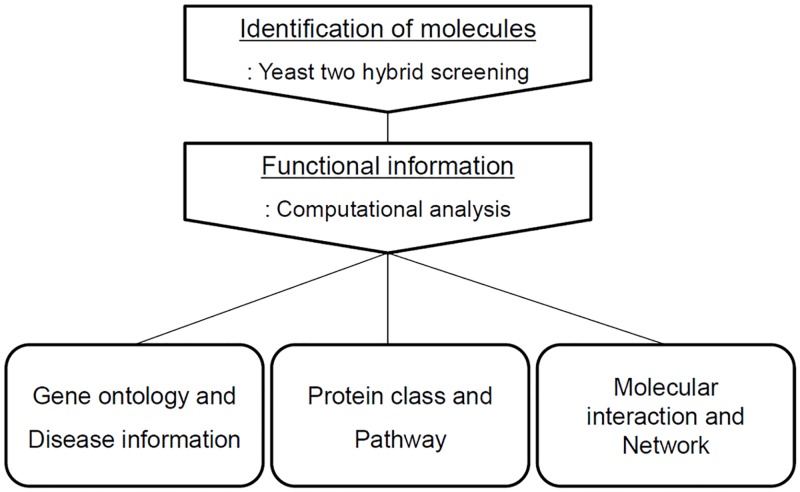
Overview of the experimental procedure. A scheme of the experimental workflow used for identification and functional analysis of calgranulin B interacting molecules was illustrated.

Calgranulin B-interacting molecules were successfully identified and various computational analyses draw functional information, which could provide the biological role of calgranulin B in the context of a complex molecular interaction network. The whole scheme of the experimental procedures was represented in Fig 1.

### Identification of calgranulin B-interacting partners using a yeast two-hybrid system

**Fig 2:**
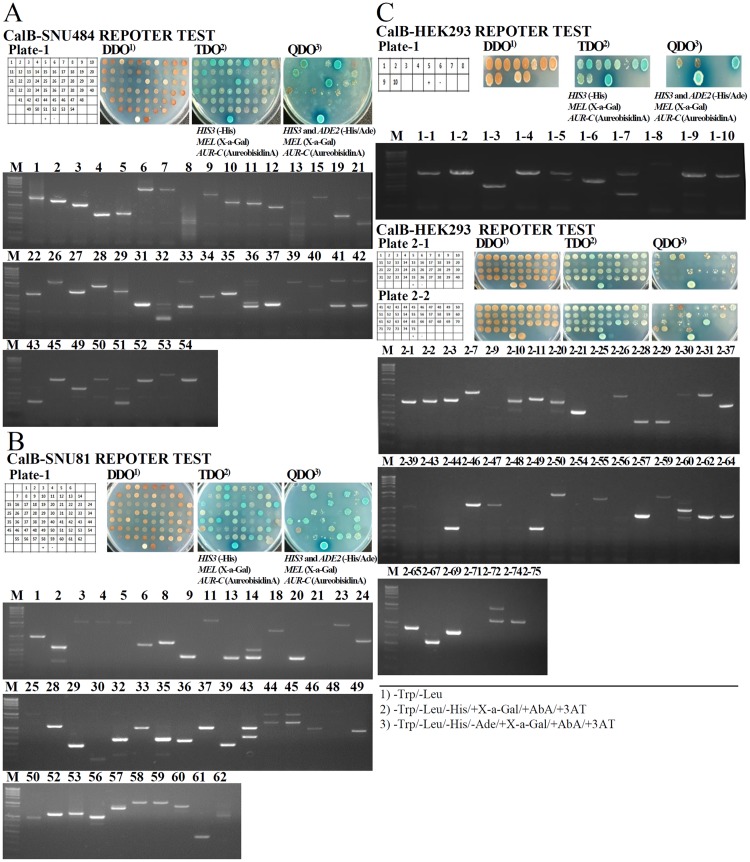
Yeast two-hybrid screening of calgranulin B-interacting partners. Positive interactions were identified through growth in selective media with different levels of restrictiveness. Positive clones were isolated and sequenced from the SNU-484 human gastric cancer cell line (A), the SNU-81 human colon cancer cell line (B), and the HEK293 human kidney cell line (C).

cDNA libraries were derived from the SNU-484 human gastric cancer cell line, the SNU-81 human colon cancer cell line, and the HEK293 normal human kidney cell line, and calgranulin B-interacting partners were screened using the bait plasmid, pGBKT7-calgranulin B. Interactions of bait and prey proteins were examined by assessing growth on two selective media with different levels of restrictiveness: TDO (-His/X-a-Gal/AbA/) and QDO (-His/-Ade/X-a-Gal/AbA) (Fig 2). Screening of 1.55x10^7^ and 1.87x10^7^ primary transformants obtained from SNU-484 and SNU-81 cells, respectively, yielded 25 and 27 positive clones, respectively (Fig 2A and 2B, upper). These clones were isolated, sequenced and aligned using the NCBI BLAST alignment search tool. We found that 14 and 13 of the clones, respectively, were in-frame; these were selected as potential candidate calgranulin B-interacting partners in gastric and colon cancers, respectively (Fig 2A and 2B, lower). We also performed two screenings in HEK293 cells, and obtained 1.76x10^7^ and 1.41x10^7^ primary transformants that yielded 10 and 34 positive clones, respectively. Four and 15 of the positive clones were found to be in-frame (Fig 2C), and were subjected to further analysis to reveal the biological meaning and relationship with diseases in the context of an interaction with calgranulin B. All of the selected candidate calgranulin B-interacting genes identified in the three cell lines by Y2H assay system are listed in Table 1.

**Table 1:** List of identified significant genes interacting with calgranulin B.

Cell line	ID	Description	Frame	Reporter expression			
				*MEL*	*AUR-C*	*HIS3*	*ADE2*
**SNU-484**	3	A-kinase-interacting protein 1 isoform b [Homo sapiens]	+1	+++	+++	+++	+++
	4	PREDICTED: thioredoxin, mitochondrial isoform X1 [Homo sapiens]	+1	+++	+++	+++	-
	7	pyruvate dehydrogenase protein X component, mitochondrial isoform 2 [Homo sapiens]	+1	+++	+++	+++	+++
	9	60S ribosomal protein L18 isoform 1 [Homo sapiens]	+1	++	++	++	+
	11	ribosomal protein L11, isoform CRA_a [Homo sapiens]	+1	+	++	++	-
	12	A-kinase-interacting protein 1 isoform b [Homo sapiens]	+1	+++	+++	+++	+++
	28	ran-binding protein 9 [Homo sapiens]	+1	+++	+++	+++	++
	29	NAG13 [Homo sapiens]	+1	+	+++	+++	++
	31	afadin isoform X17 [Homo sapiens]	+1	++	+++	++	+++
	34	peptidyl-prolyl cis-trans isomerase FKBP2 precursor [Homo sapiens]	+1	++	+++	+++	++
	35	procollagen C-endopeptidase enhancer 1 precursor [Homo sapiens]	+1	+++	+++	+++	+
	37	ribosomal L1 domain-containing protein 1 [Homo sapiens]	+1	+	+++	+++	++
	39	dystonin, isoform CRA_f [Homo sapiens]	+1	+	++	++	-
	50	COMM domain-containing protein 1 [Homo sapiens]	+1	++	++	++	-
**SNU-81**	1	succinate dehydrogenase [ubiquinone] iron-sulfur subunit, mitochondrial precursor [Homo sapiens]	+1	+++	+++	+++	+++
	5	probable tRNA pseudouridine synthase 1 [Homo sapiens]	+1	+	++	++	-
	6	Homo sapiens chromosome 19, alternate assembly CHM1_1.1	+1	+++	+++	+++	-
	18	NEDD4-like E3 ubiquitin-protein ligase WWP2 isoform WWP2-N [Homo sapiens]	+1	++	++	++	-
	24	60S ribosomal protein L39 [Homo sapiens]	+1	+++	+++	+++	+++
	46	OCIA domain containing 2, isoform CRA_b [Homo sapiens]	+1	+++	+++	+++	+++
	49	peptidyl-prolyl cis-trans isomerase FKBP2 precursor [Homo sapiens]	+1	++	++	++	++
	50	OCIA domain-containing protein 2 isoform 2 [Homo sapiens]	+1	+++	+++	+++	+++
	52	integrin beta-1-binding protein 1 isoform 1 [Homo sapiens]	+1	++	+	+	-
	57	PREDICTED: lysophosphatidic acid phosphatase type 6 isoform X1 [Homo sapiens]	+1	++	++	++	++
	58	fructose-bisphosphate aldolase C [Homo sapiens]	+1	++	++	++	+
	60	sodium/potassium-transporting ATPase subunit beta-1 [Homo sapiens]	+1	+++	+++	+++	+++
	62	signal peptidase complex subunit 2 [Homo sapiens]	+1	+++	+++	+++	+++
**HEK293**	1–2	KIAA1109 protein	+1	++	+++	+++	++
	1–4	KIAA1109 protein	+1	++	+++	+++	++
	1–6	splicing factor, arginine/serine-rich 2, interacting protein, isoform CRA_b	+1	+++	+++	+++	-
	1–8	zinc finger protein 737 isoform X3	+1	+++	+++	+++	+++
	2–1	rho GDP-dissociation inhibitor 1 isoform a [Homo sapiens]	+1	+++	+++	+++	++
	2–3	DNA replication licensing factor MCM7 isoform 2 [Homo sapiens]	+1	+++	+++	+++	+++
	2–4	DNA replication licensing factor MCM7 isoform 2 [Homo sapiens]	+1	+++	+++	+++	+++
	2–7	AT-rich interactive domain-containing protein 2 isoform X1	+1	++	+++	+++	+++
	2–11	differentially expressed in FDCP 8 homolog isoform 2 [Homo sapiens]	+1	++	+++	+++	++
	2–28	DHX30 protein [Homo sapiens]	+1	+++	+++	+++	+
	2–31	reticulon-4 receptor precursor [Homo sapiens]	+1	++	+++	+++	+
	2–37	60S ribosomal protein L12 [Homo sapiens]	+1	+	++	++	-
	2–50	NOMO3 protein, partial [Homo sapiens]	+1	+++	+++	+++	+++
	2–55	ran-binding protein 10 isoform X1 [Homo sapiens]	+1	+++	+++	+++	+++
	2–57	peroxiredoxin-6 [Homo sapiens]	+1	++	++	++	+
	2–59	CEV14 [Homo sapiens]	+1	++	+++	+++	++
	2–60	PREDICTED: COP9 signalosome complex subunit 1 isoform X13 [Homo sapiens]	+1	++	++	++	-
	2–62	60S ribosomal protein L12 [Homo sapiens]	+1	++	++	++	++
	2–65	probable hydrolase PNKD isoform 3 precursor [Homo sapiens]	+1	++	++	++	+

### Gene ontology and top diseases information

**Fig 3:**
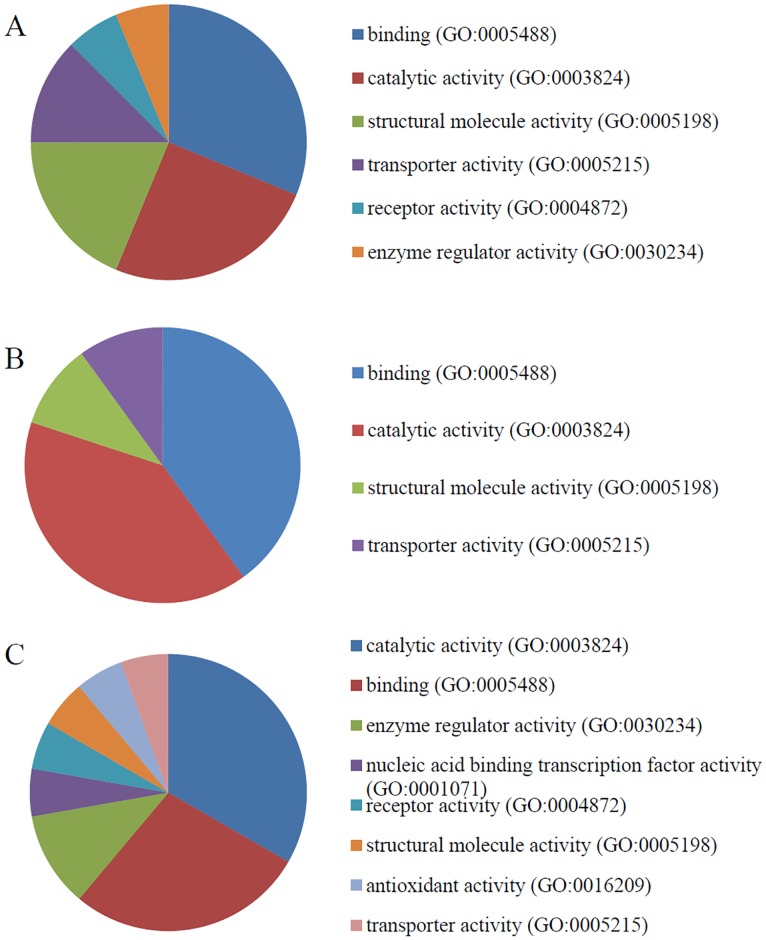
Molecular function gene ontology (GO) terms overrepresented among the calgranulin B-interacting partners. The distributions of the molecular functions represented by the calgranulin B-interacting molecules identified in SNU-484 (A), SNU-81 (B), and HEK293 (C) cells are shown.

**Fig 4:**
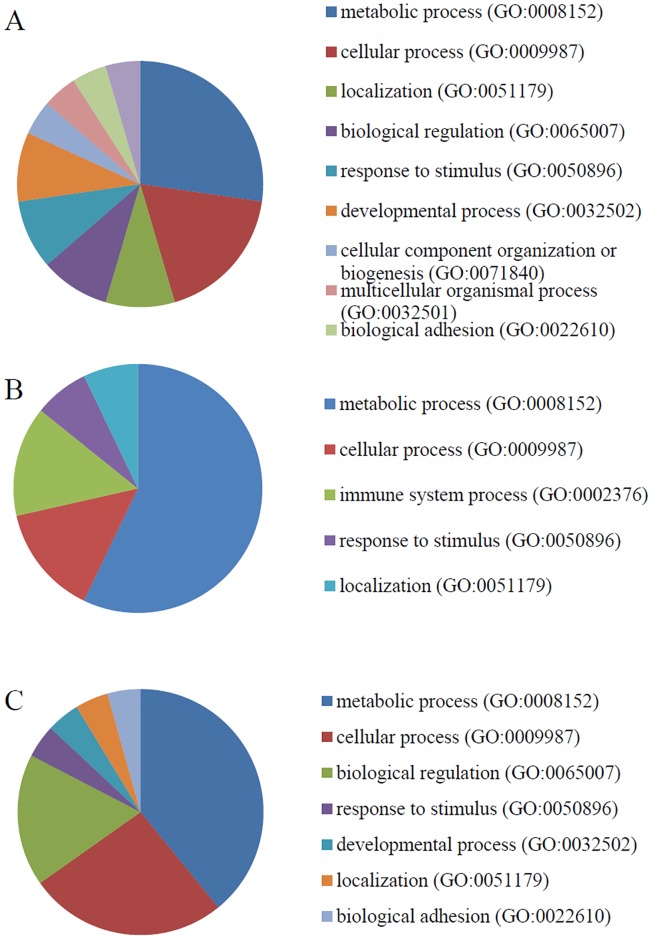
Biological processes overrepresented among the calgranulin B-interacting partners. The distributions of the biological processes represented by the calgranulin B-interacting molecules identified in SNU-484 (A), SNU-81 (B), and HEK293 (C) cells are shown.

**Fig 5:**
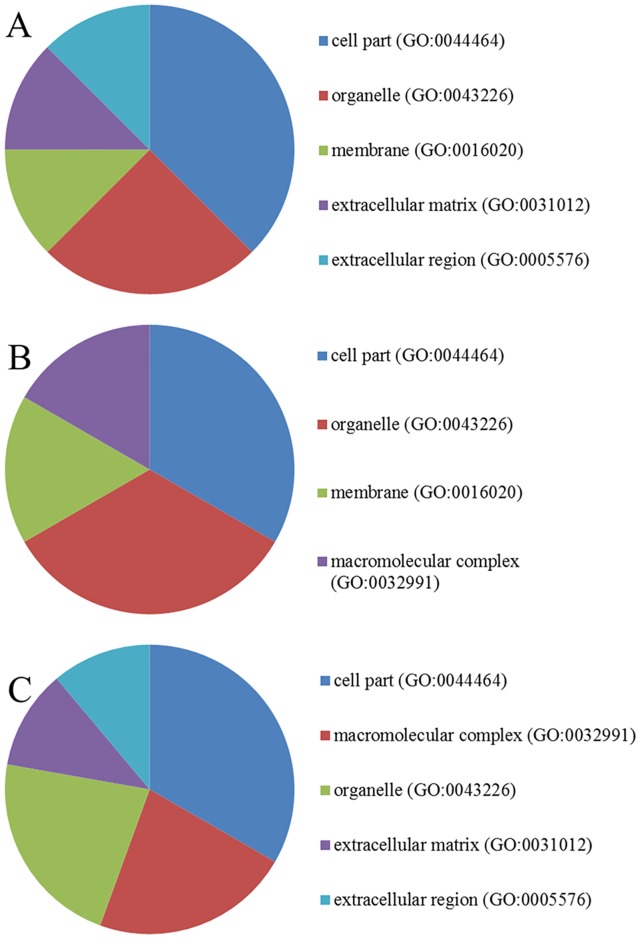
Cellular components associated with the calgranulin B-interacting molecules. The calgranulin B-interacting molecules identified in SNU-484 (A), SNU-81 (B), and HEK293 (C) cells were categorized according to cellular component information.

PANTHER was used to identify the GO terms associated with the molecular function, biological activity, and cellular component(s) associated with each candidate calgranulin B-interacting protein. The results of this analysis (Figs 3–5) revealed that calgranulin B appears to be associated with slightly different molecular functions, biological processes and cellular components in SNU-484, SNU-81, and HEK293 cells. However, in all three lines, binding (GO:0005488) and catalytic activity (GO:0003824) were the most highly represented molecular functions, with representations of 31.3% and 33.3% in SNU-484 and HEK293 cells, respectively and 40% for both cases in SNU-81 cells. The next most common functions were structural molecule activity (GO:0005198) in SNU-484 cells, enzyme regulation (GO:0030234) in HEK293 cells, and structural molecule activity (GO:0005198) and transportation (GO:00052158) in SNU-81 cells (Fig 3).

The calgranulin B-interacting molecules were also classified according to their related biological activities/processes (i.e., recognized series of events or molecular functions; www.geneontology.org). In all three cell lines, the most highly represented biological process was metabolic process (GO:0008152), followed by cellular processes (GO:0009987) in SNU-484 and HEK293 cells and immune processes (GO:0002376) in SNU-81 cells (Fig 4).

The cellular component portion of the GO analysis was used to classify the candidate proteins by their known locations at the levels of subcellular structure and macromolecular complexes (www.geneontology.org). Our results revealed that the calgranulin B-interacting molecules were largely localized in the cell part (GO:0044464) and organelle (GO:0043226) components of SNU-484 and SNU-81 cells, while they were distributed to the cell part (GO: 0044464), macromolecular complex (GO: 0032991) and organelle (GO: 0043226) components of HEK293 cells (Fig 5).

Ingenuity Pathway Analysis (IPA) was used to identify the most highly represented diseases and disorders among the calgranulin B-interacting proteins of each cell line. Cancer was the most highly represented disease/disorder in SNU-81 and HEK293 cells, whereas cardiovascular disease was the most highly represented disease/disorder in SNU-484 cells. The full results of this analysis are presented in Table 2.

**Table 2:** List of diseases and disorders associated with molecules identified in three cell lines.

Cell line	Name	p-value	# molecules
**SNU-484**	Cardiovascular Disease	6.45E-04–6.45E-04	1
	Connective Tissue Disorders	6.45E-04–6.45E-04	1
	Dermatological Diseases and Conditions	6.45E-04–6.45E-04	1
	Developmental Disorder	6.45E-04–6.45E-04	2
	Hematological Disease	1.35E-02–6.45E-04	2
**SNU-81**	Cancer	4.91E-02–5.91E-04	5
	Connective Tissue Disorders	1.41E-02–5.91E-04	3
	Developmental Disorder	1.41E-02–5.91E-04	2
	Gastrointestinal Disease	3.49E-03–5.91E-04	2
	Hereditary Disorder	1.41E-02–5.91E-04	2
**HEK 293**	Cancer	4.13E-02–8.60E-04	3
	Developmental Disorder	3.47E-02–8.60E-04	2
	Hereditary Disorder	4.13E-02–8.60E-04	2
	Infectious Diseases	8.60E-02–8.60E-04	1
	Neurological Disease	3.55E-02–8.60E-04	4

### Protein classes and pathways related to the identified calgranulin B-interacting partners

**Fig 6:**
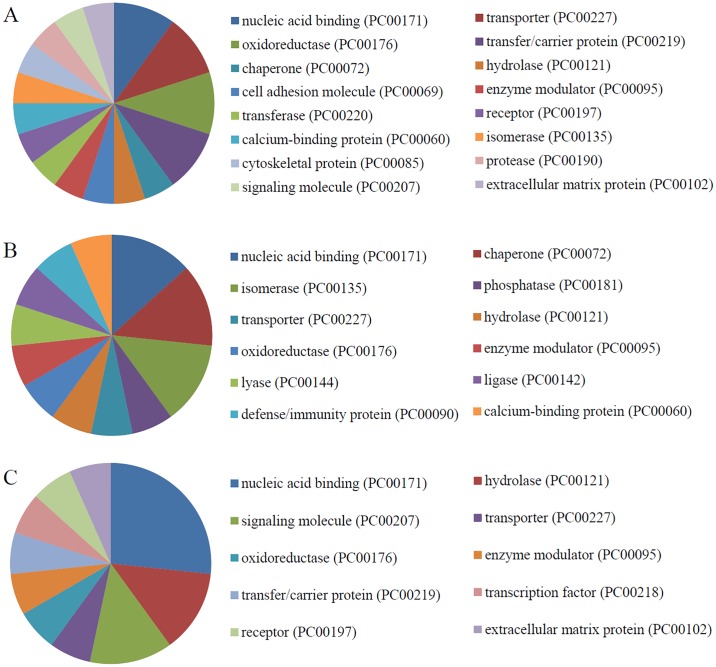
PANTHER analysis of protein classes overrepresented among the calgranulin B-interacting partners. Protein class distributions of the calgranulin B-interacting molecules identified in SNU-484 (A), SNU-81 (B), and HEK293 (C) cells are shown.

The protein class distribution in each cell line was evaluated by PANTHER analysis (Fig 6). Our results revealed that nucleic acid binding (PC00171) was the most highly represented protein class in all three cell lines, followed by: transporter (PC00227), oxidoreductase (PC00176), and transfer/carrier (PC00219) functions in SNU-484 cells (Fig 6A); chaperone (PC00072) and isomerase (PC00135) functions in SNU-81 cells (Fig 6B); and hydrolase (PC00121) and signaling (PC00207) functions in HEK293 cells (Fig 6C).

**Fig 7:**
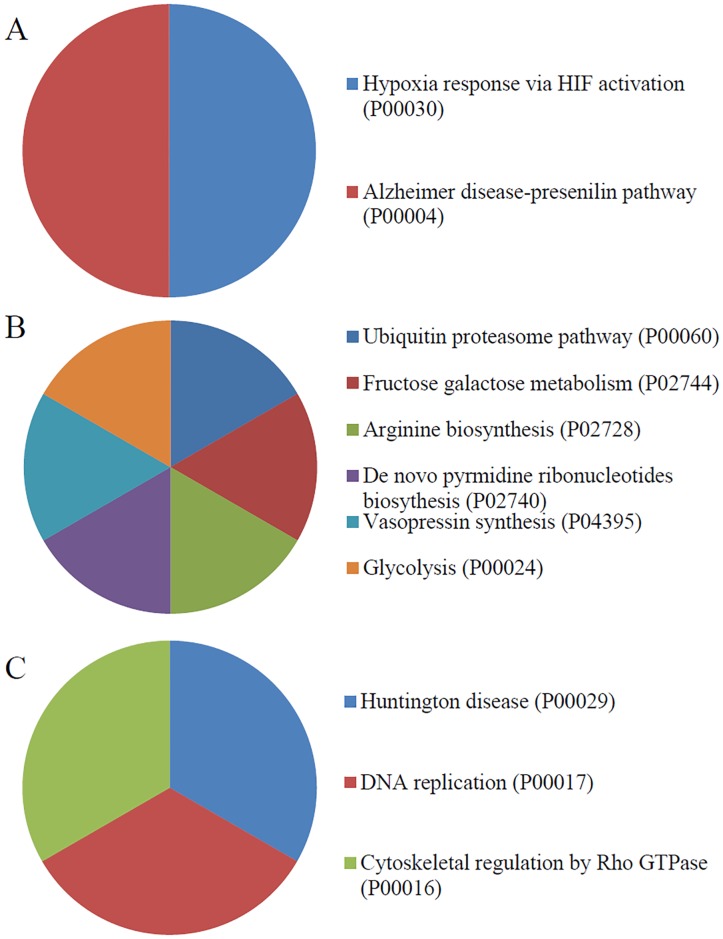
PANTHER analysis of pathways overrepresented among the calgranulin B-interacting partners. The pathway distributions of the calgranulin B-interacting molecules identified in SNU-484 (A), SNU-81 (B), and HEK293 (C) cells are shown.

PANTHER pathway analysis was used to identify pathways that were highly represented among the calgranulin B-interacting partners identified in SNU-484, SNU-81, and HEK293 cells. Interestingly, the results differed among the three cell lines. In SNU-484 cells, the calgranulin B-interacting partners were often involved in the Alzheimer disease-presenilin pathway (P00004), the hypoxia response via HIF activation (P00030) (Fig 7A). In SNU-81 cells, the overrepresented pathways included the ubiquitin proteasome pathway (P00060), the fructose-galactose metabolism pathway (P02744), and the arginine biosynthesis pathway (P02728), the de novo pyrimidine ribonucleotides biosynthesis (P02740), Vasopressin synthesis (P04395), and Glycolysis (P00024) (Fig 7B). In HEK293 cells, the overrepresented pathways included those related to Huntington disease (P00029), DNA replication (P00017), and Cytoskeletal regulation by Rho GTPase (P00016) (Fig 7C).

### Molecular network analysis and cancer-related calgranulin B-interacting proteins

**Fig 8:**
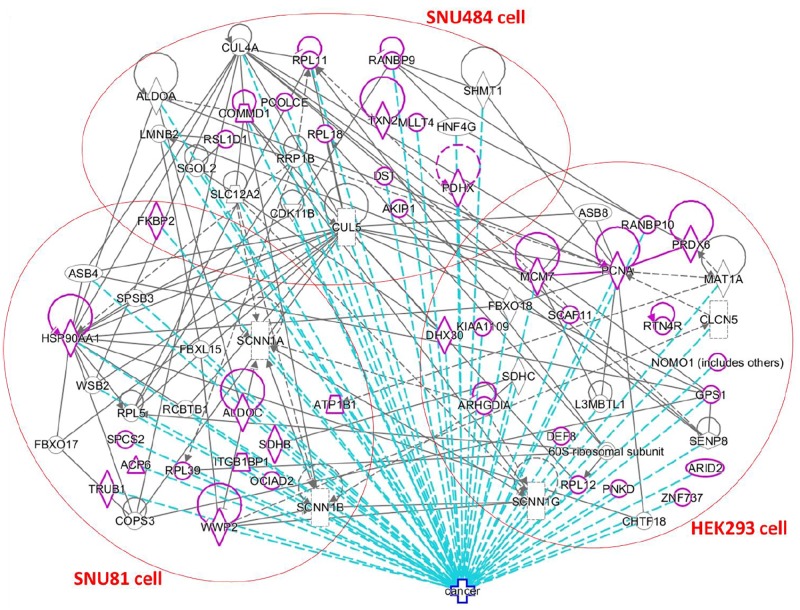
Network analysis and cancer associations of the calgranulin B-interacting molecules. In the generated network, molecular interactions are shown by grey lines, the identified calgranulin B-interacting molecules are highlighted in purple, and molecules that have been associated with cancer are indicated by dotted blue lines.

To obtain a more comprehensive view of the molecular network related to the calgranulin B-interacting molecules identified herein, we used IPA to analyze their direct and indirect molecular interactions. The results are shown in Fig 8, where molecular interactions are indicated with grey lines, the identified calgranulin B-interacting molecules are highlighted in purple, the relevant cell lines are indicated in red circles, and the 46 calgranulin B-interacting molecules that have been associated with cancer are indicated with dotted blue lines. All candidate molecules interacting with calgranulin B were unique in three cell lines except FKBP2 that was found in two cancer cell lines, SNU-484 and SNU-81. However molecular network analysis revealed they had direct and/or indirect interaction across 3 cell lines and most of them were involved in cancer disease development.

### Calgranulin B-interacting proteins associated with the extracellular space and plasma membrane

**Fig 9:**
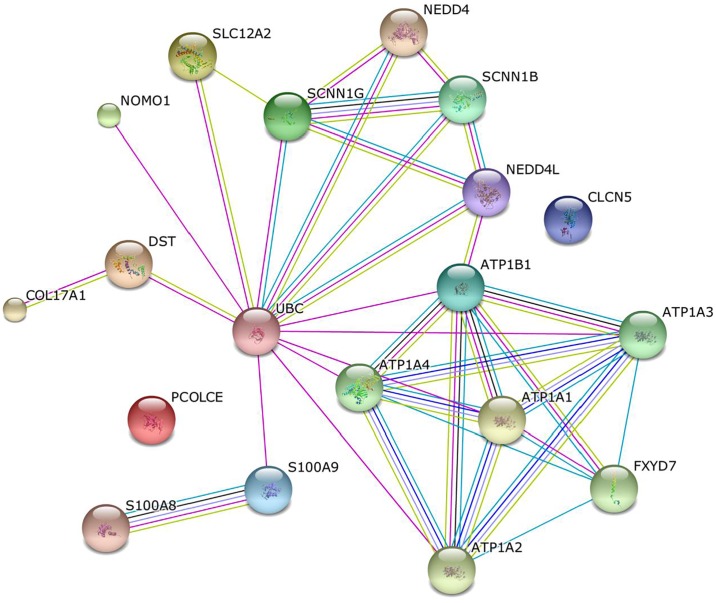
Communication of calgranulin B with extracellular environment via polyubiquitin-C(UBC) in SNU-484, SNU-81, and HEK293 cells. Direct interactions with calgranulin B (S100A9) were predicted using STRING analysis, and are indicated by different colored lines: green, red, blue, black, purple, light blue, yellow, sky blue based on the types of evidence for associations.

Among the identified calgranulin B-interacting proteins, those known to be localized to the extracellular space and plasma membrane were further characterized by network analysis to reveal interactive relationship with calgranulin B. As shown in Fig 9, calgranulin B (S100A9) was found to directly interact with polyubiquitin-C (encoded by *UBC*), which is known to directly interact with various other calgranulin B-interacting partners, including dystonin (encoded by *DST*) in SNU-484 cells, ATPase subunit beta-1 (ATP1B1, encoded by *ATP1B1*) in SNU-81 cells, and nodal modulator 1 (encoded by *NOMO1*) in HEK293 cells.

## Discussion

Yeast two-hybrid screening analysis is a molecular biology technique that enables researchers to uncover protein-protein and protein-DNA interactions by testing for physical interactions between relevant fragments. Y2H screening has been steadily used to identify new interacting partners for a protein of interest, or they may confirm binary protein-protein interactions, which play crucial roles in almost all biological processes [18]. Here, we used Y2H analysis to identify candidate calgranulin B-interacting molecules (Fig 2), and subjected these proteins to PANTHER analysis of: their predicted activities, which emphasized binding and catalytic activity (Figs 3 and 6); their cellular involvements, which emphasized metabolic and cellular processes (Fig 4); and their cellular localizations (Fig 5). Molecular function, biological activity and cellular components of identified molecules showed slight differences among three cell lines but most terms were common in all three cell lines tested. To determine further biological characteristics of calgranulin B-interacting proteins, previously reported calgranulin B-interacting molecules on the BioGRID database (http://thebiogrid.org) were analyzed by PANTHER and found to have correlation with the present data. For example, the most highly represented molecular function; cellular component, biological process, and protein class of the previously reported 92 molecules are same with our present study as binding, cell part, metabolic process, and nucleic acid binding, respectively (data not shown).

However, signaling pathways involved calgranulin B interacting partners were totally different in each cell line (Fig 7) providing an involvement of unique signaling cascades. Ubiquitin proteasome pathway represented in SNU-81 colon cell is very interesting considering internalization pathway of calgranulin B in colon cancer cells. Ubiquitination, one of the post-translational modifications of proteins can lead to targeting for degradation by the proteasome machinery as well as can serve as a sorting signal for regulation of internalization such as activation of receptor endocytosis [19,20]. In addition, ubiquitination of integral plasma membrane proteins triggers their rapid internalization into endocytic pathway [21]. Recently we found that calgranulin B released from immune cells can be internalized specifically into colon cancer cells and suppressed cell proliferation [22] but the molecular mechanism of internalization pathway was not clear. Ubiquitin proteasome pathway proposed in this study could provide a possible mechanism of calgranulin B internalization in colon cancer cells.

Among the calgranulin B-interacting molecules known to be involved in cancer (Fig 8, purple), FKBP2 (FK506-binding protein 2) was identified from both SNU-484 and SNU-81 cells. FKBP2, which is a member of the immunophilin protein family, contributes to immunoregulation and is thought to function as an ER chaperone and a component of membrane cytoskeletal scaffolding [23]. The FKBP2 gene has been associated with pathological osteoporosis [24], and its protein product was suggested to be associated with the development of type 2 diabetes [25]. Northern blotting revealed that FKBP2 is expressed in tissues that are predisposed to hyperplasia in multiple endocrine neoplasia type 1 (MEN1) patients; however, mutation analysis of MEN1 kindreds and sporadic tumors excluded FKBP2 as a candidate gene for MEN1 [26]. No previous report has suggested any molecular cooperation between FKBP2 and calgranulin B. Here, we show for the first time that calgranulin B may have FKBP2-mediated function in gastrointestinal cancer cells.

No previous study has exhibited a direct link between polyubiquitin-C (encoded by *UBC*) and cancer. However, many studies have shown that polyubiquitin-C interacts with various cancer-associated effector molecules, including cdk1 [27], E2F1 [28], epidermal growth factor receptor (EGFR) [29], HDAC3 [27], HIF1A [30], Mdm2 [31], NOTCH1 [32], and p53 [29]. Our present results with previous proteome analysis reveal that polyubiquitin-C interacts with calgranulin B [33–35], suggesting that polyubiquitin-C may act as an important mediator that connects calgranulin B to various proteins in the extracellular space and plasma membrane, such as Na-K-Cl cotransporter 1 (NKCC1, encoded by *SLC12A2*) and dystonin (encoded by *DST*) in SNU-484 cells, and ATPase subunit beta-1 (ATP1B1, encoded by *ATP1B1*) in SNU-81 cells (Fig 9). These interactions with polyubiquitin-C have been reported in the previous studies [34,36–39], which suggest that calgranulin B may communicate with the tumor microenvironment via polyubiquitin-C.

NKCC1 is a membrane-transport protein that governs the active bi-directional transport of sodium, potassium, and chloride [40]. NKCC1 expression has been shown to predict poor prognosis in lung adenocarcinoma [41] and affect the G2/M checkpoint in esophageal squamous cell carcinoma [42]. In glioma cells, inhibition of NKCC1 was found to reduce invasion [43,44] and augment temozolomide-induced apoptosis [45]. Blockade of NKCC1 function also diminished the proliferation of poorly differentiated human gastric cancer cells by affecting G0/G1 phase [42]. It is not yet known whether the interaction between calgranulin B and NKCC1 blocks or augments the function of NKCC1, but studies showing that calgranulin B and calprotectin exert pro-apoptotic effects [46–48] may suggest that calgranulin B inhibits the activity of NKCC1 in gastric cancer cells.

Dystonin, which is a neural isoform of bullous pemphigoid antigen 1, has an N-terminal actin-binding domain and is essential for maintaining the cytoskeletal integrity of neurons [49]. No previous study has linked dystonin to gastric cancer. Further work is needed to clarify the potential for calgranulin B to communicate with the extracellular environment via dystonin.

Among the identified proteins, ATPase subunit beta-1 is unique in that it may interact with calgranulin B to facilitate colon cancer progression (Fig 8) and/or communicate directly with polyubiquitin-C (Fig 9). ATPase is a well-known membrane protein that is responsible for maintaining the electrochemical gradients of Na and K ions across the plasma membrane. It can also be a target of TGF-beta1 during TGF-beta1-induced epithelial-to-mesenchymal transition (EMT) [50], and participate in the drug sensitization of cancer cells [51]. In contrast to the interaction between calgranulin B and NKCC1 we do not expect to see a calgranulin B-mediated change in ATPase activity. However, our overall data demonstrate that ATPase subunit beta-1 is a most promising candidate for future efforts to study the potential function of calgranulin B in colon cancer cells.

In sum, we herein screened for calgranulin B-interacting partners and found that they included both cytosolic and plasma membrane proteins. The molecular linkages identified herein suggest that calgranulin B may have multiple anti-tumor functions acting against the progression of gastrointestinal cancer. Our novel findings suggest that further functional studies are warranted to investigate the signaling downstream of the encounters between calgranulin B and its interacting partners.
